# Toxin Mediates Sepsis Caused by Methicillin-Resistant *Staphylococcus epidermidis*

**DOI:** 10.1371/journal.ppat.1006153

**Published:** 2017-02-02

**Authors:** Li Qin, Fei Da, Emilie L. Fisher, Daniel C. S. Tan, Thuan H. Nguyen, Chih-Lung Fu, Vee Y. Tan, Joshua W. McCausland, Daniel E. Sturdevant, Hwang-Soo Joo, Shu Y. Queck, Gordon Y. C. Cheung, Michael Otto

**Affiliations:** 1 Pathogen Molecular Genetics Section, Laboratory of Bacteriology, National Institute of Allergy and Infectious Diseases, The National Institutes of Health, Bethesda, Maryland, United States of America; 2 Department of Dermatology, Wuhan No.1 Hospital, Tongji Medical College, Huazhong University of Science and Technology (HUST), Wuhan, China; 3 Department of Pharmacology, School of Pharmacy, Fourth Military Medical University, Xi’an, China; 4 Research Technology Branch, Rocky Mountain Laboratories, National Institute of Allergy and Infectious Diseases, The National Institutes of Health, Hamilton, Montana, United States of America; Imperial College London, UNITED KINGDOM

## Abstract

Bacterial sepsis is a major killer in hospitalized patients. Coagulase-negative staphylococci (CNS) with the leading species *Staphylococcus epidermidis* are the most frequent causes of nosocomial sepsis, with most infectious isolates being methicillin-resistant. However, which bacterial factors underlie the pathogenesis of CNS sepsis is unknown. While it has been commonly believed that invariant structures on the surface of CNS trigger sepsis by causing an over-reaction of the immune system, we show here that sepsis caused by methicillin-resistant *S*. *epidermidis* is to a large extent mediated by the methicillin resistance island-encoded peptide toxin, PSM-mec. PSM-mec contributed to bacterial survival in whole human blood and resistance to neutrophil-mediated killing, and caused significantly increased mortality and cytokine expression in a mouse sepsis model. Furthermore, we show that the PSM-mec peptide itself, rather than the regulatory RNA in which its gene is embedded, is responsible for the observed virulence phenotype. This finding is of particular importance given the contrasting roles of the *psm-mec* locus that have been reported in *S*. *aureus* strains, inasmuch as our findings suggest that the *psm-mec* locus may exert effects in the background of *S*. *aureus* strains that differ from its original role in the CNS environment due to originally “unintended” interferences. Notably, while toxins have never been clearly implied in CNS infections, our tissue culture and mouse infection model data indicate that an important type of infection caused by the predominant CNS species is mediated to a large extent by a toxin. These findings suggest that CNS infections may be amenable to virulence-targeted drug development approaches.

## Author Summary

Coagulase-negative staphylococci (CNS) are the leading cause of sepsis in hospitalized patients, causing a significant number of deaths. This situation is further worsened by a limitation of therapeutic options due to the fact that most CNS infectious isolates are resistant to methicillin. CNS sepsis has been assumed to be due to on over-reacting immune response triggered by invariant bacterial surface structures. By using tissue culture and animal infection model-based evidence, we here show that in contrast to that notion, the PSM-mec toxin produced by methicillin-resistant strains of the leading CNS species *Staphylococcus epidermidis* has a strong impact on the severity of sepsis and its outcome. This is the first report to link a toxin to the pathogenesis of the most frequent bacterial cause of sepsis. Notably, these findings pave the way for anti-virulence strategies against this widespread and deadly type of infection.

## Introduction

Bacterial sepsis is a frequent cause of death in hospitalized patients. Coagulase-negative staphylococci (CNS) are the leading cause of nosocomial sepsis, especially in neonates [1–3]. CNS sepsis most often originates from the infection of indwelling medical devices, such as in catheter-related bloodstream infections (CRBSIs) or central line-associated blood stream infections (CLABSIs) [4]. Most prominent among CNS infections are those due to the skin commensal *Staphylococcus epidermidis* [5]. However, the bacterial factors contributing to the development of sepsis, in particular in CNS, are poorly understood.

Given that toxins have long been assumed to be widely absent from CNS [6], sepsis caused by *S*. *epidermidis* and other CNS, similar to other Gram-positive bacteria, has so far been believed to be due predominantly to an overwhelming immune reaction directed against invariable, pro-inflammatory cell surface molecules, such as teichoic acids and lipopeptides [7]. Recently, the notion that CNS do not commonly produce toxins had to be revised with the discovery of the pro-inflammatory and cytolytic phenol-soluble modulin (PSM) staphylococcal toxin family [8]. However, due to the difficulties associated with genetic manipulation of *S*. *epidermidis* and other CNS, the roles of PSMs in CNS infections, including most notably sepsis, have hitherto remained unexplored.

Most *S*. *epidermidis* blood infections are caused by methicillin-resistant strains (MRSE), with methicillin resistance rates even exceeding those found among *S*. *aureus* [9]. Methicillin resistance is encoded on so-called staphylococcal chromosome cassette (SCC) *mec* mobile genetic elements, which are believed to have originated from CNS, from where they were transferred to *S*. *aureus* [10]. While other PSMs are core-genome encoded [8], one PSM toxin, called PSM-mec, is encoded within SCC*mec* elements of subtypes II, III, and VIII [11, 12]. The *psm-mec* gene is embedded in a short regulatory (sr) RNA, which in *S*. *aureus* has been reported to down-regulate the production of other PSMs and thereby decrease virulence [13, 14]. While this effect has been claimed to generally explain lower virulence of hospital-associated as compared to community-associated MRSA strains [13], it is quite moderate and extensively strain-dependent [11, 13]. Recently, the *psm-mec* locus has been introduced on a plasmid into some CNS that naturally lack *psm-mec*, and was reported to trigger gene regulatory changes [15]; but the roles that the PSM-mec peptide or the *psm-mec* srRNA naturally play in CNS including *S*. *epidermidis* are unknown.

Here we analyzed the role of the *psm-mec* locus in *S*. *epidermidis* sepsis by using tissue culture and animal infection models. Our findings show for the first time that a toxin can have a strong impact on CNS sepsis, setting the stage for anti-virulence strategies directed against this frequent and deadly infection.

## Results and Discussion

To analyze the impact of the *psm-mec* locus on *S*. *epidermidis* sepsis, we produced isogenic *psm-mec* deletion mutants (Δ*psm-mec*) in two MRSE strains, a clinical isolate (SE620) and the genome-sequenced strain RP62A. PSM-mec production in these strains is representative of clinical PSM-mec-positive MRSE (**S1 Fig**), which we determined in a clinical *S*. *epidermidis* strain collection from Norway to occur in ~ 2/3 (59/91) of the ~ 50% (91/180) methicillin-resistant *S*. *epidermidis*. We also introduced a point mutation in the start codon of the *psm-mec* gene in the genome of strain SE620 to differentiate between effects mediated by the PSM-mec peptide versus those due to the *psm-mec* srRNA (psm-mec*). Notably, the stability of the *psm-mec* RNA was not significantly altered by introduction of the 1-basepair start codon mutation (**S2 Fig**).

**Fig 1:**
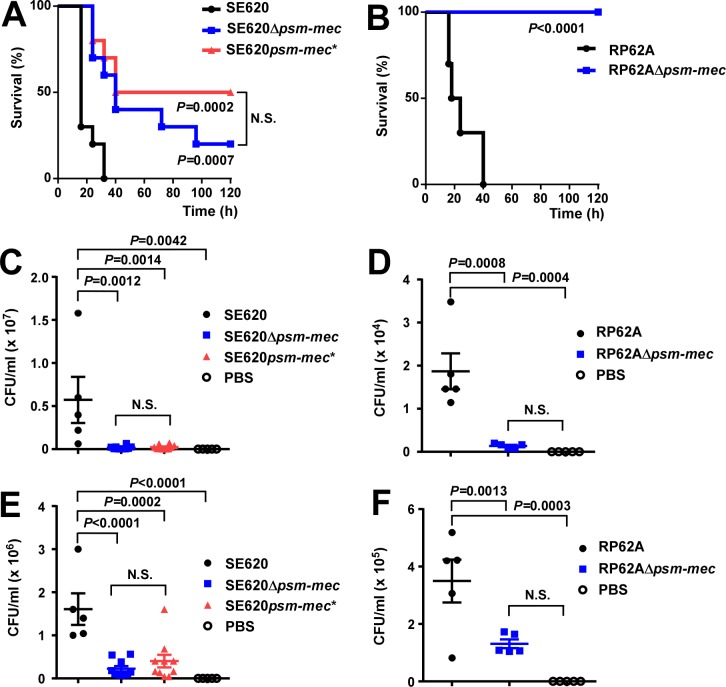
Mouse sepsis model. Female, 6–10 weeks old, C57BL/6NCRl mice (n = 5 for all groups except SE620Δ*psm-mec* and SE620psm-mec*, n = 10) were injected via the tail vein with 5 x 10^8^ CFU of the indicated bacterial strains and monitored for disease development every 8 h for up to 120 h. (**A,B**) Survival curves; (**C,D**) CFU in blood at 12 h; (**E,F**) CFU in kidneys at 12 h. Statistical analysis is by Log-rank (Mantel-Cox) tests for survival curves, otherwise using 1-way ANOVA with Bonferroni post-tests. Error bars show ±SEM. N.S., not significant. Δ*psm-mec*, isogenic *psm-mec* deletion mutant; psm-mec*, *psm-mec* gene start codon mutant.

We first analyzed those mutants in a murine sepsis model. Mortality was significantly reduced in the Δ*psm-mec* mutants of both strains (**Fig 1A and 1B**). There was no significant difference between the Δ*psm-mec* mutant and the psm-mec* start codon mutant (**Fig 1A**). Furthermore, CFU in the blood and the kidneys were strongly reduced in the Δ*psm-mec* mutants of both strains and the psm-mec* start codon mutant (**Fig 1C–1F)**. These results demonstrate a strong contribution of the PSM-mec toxin to bacteremia and mortality due to *S*. *epidermidis* sepsis, while the *psm-mec* srRNA did not show any impact.

We showed previously that synthetic PSM-mec peptide is strongly pro-inflammatory and has moderate to strong cytolytic capacity [12]. To analyze the contribution that the *psm-mec* locus has to pro-inflammatory and cytolytic capacity in the *S*. *epidermidis* background, we measured cytokine concentrations during experimental murine sepsis and determined cytolytic capacity of the bacterial strains toward human neutrophils in vitro. Cytokine concentrations during sepsis are the result of a systemic reaction due to several immune cell types, and are thus best determined in vivo, while cytolytic capacity can be most accurately measured in vitro.

**Fig 2:**
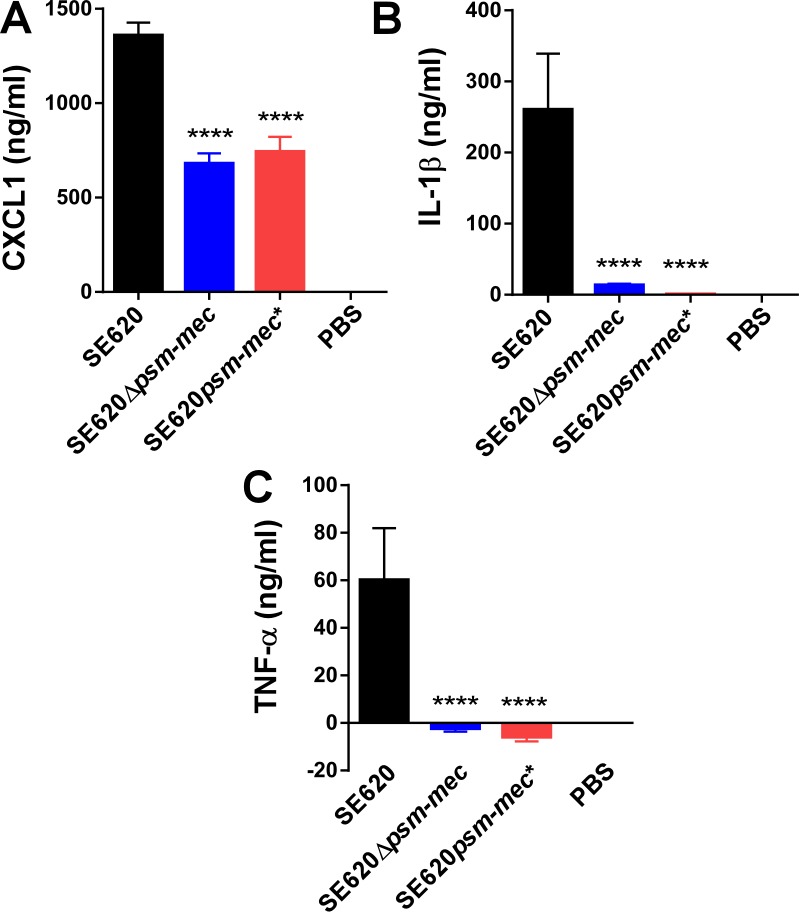
Mouse sepsis model, cytokine concentrations, strain SE620. Cytokine concentrations (**A**, CXCL1, **B**, IL-1β, **C**, TNF-α) were determined at 12 h post infection in the mouse sepsis model using commercial ELISA kits. Statistical analysis is by 1-way ANOVA with Bonferroni post-tests. Error bars show ±SEM. ****, *P*<0.0001. Δ*psm-mec*, isogenic *psm-mec* deletion mutant; psm-mec*, *psm-mec* gene start codon mutant.

**Fig 3:**
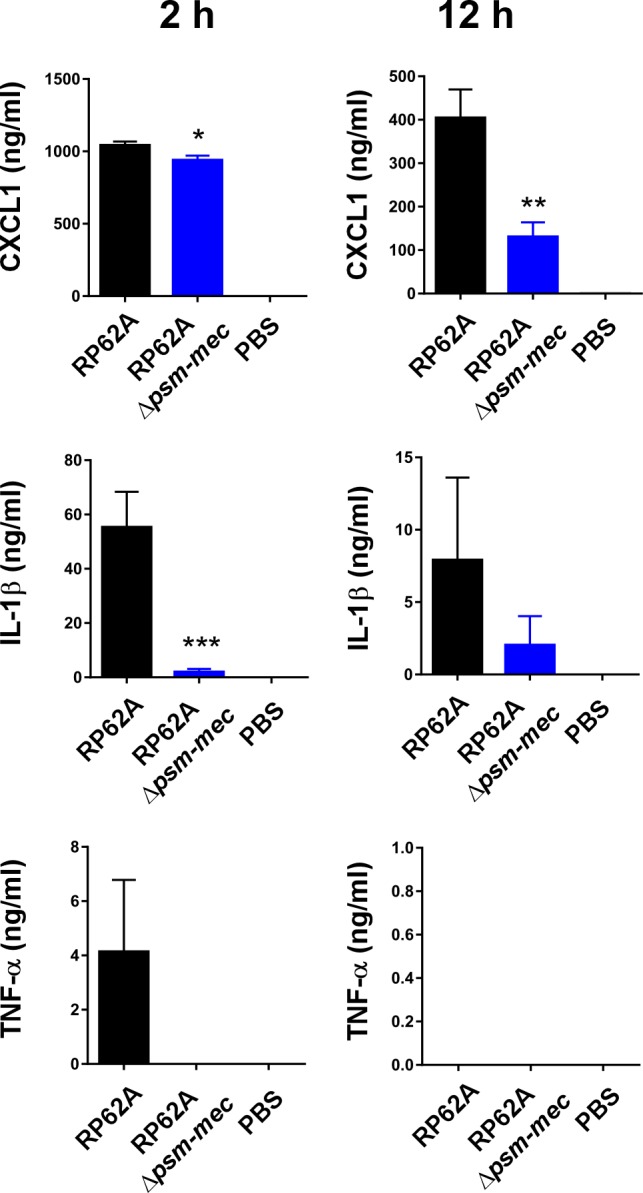
Mouse sepsis model, cytokine concentrations, strain RP62A. Cytokine concentrations in the blood of mice infected with RP62A or its *psm-mec* deletion mutant at 2 and 12 h after infection. Statistical analysis is by 1-way ANOVA; multiple comparisons using Bonferroni post-tests. Error bars show ±SEM. *, P<0.05; **, P<0.01; ***, P<0.001. Statistical results are only shown for the RP62A versus *psm-mec* mutant comparison. Note TNF-α concentrations were below the detection limit at 12 h. Δ*psm-mec*, isogenic *psm-mec* deletion mutant; psm-mec*, *psm-mec* gene start codon mutant.

The PSM-mec peptide, but not the *psm-mec* srRNA, had a strong and significant impact on the production of cytokines during murine sepsis (**Fig 2**). At 12 h after infection, the mouse IL-8 homologue CXCL1 was significantly reduced when mice were infected with the Δ*psm-mec* or psm-mec* start codon mutant of strain SE620, to about half the concentration measured in mice infected with the wild-type strain (**Fig 2A**). Concentrations of IL-1β and TNF-α were even more strongly reduced to levels not significantly different from those measured in mock (PBS) infected animals (**Fig 2B and 2C**). In the RP62A background, the phenotypes were similar, with differences being more pronounced during earlier stages of the infection (measured at 2 versus 12 h) (**Fig 3**). These results showed that the cytokine storm that commonly accompanies bacterial sepsis is strongly dependent on the PSM-mec toxin in *S*. *epidermidis*.

**Fig 4:**
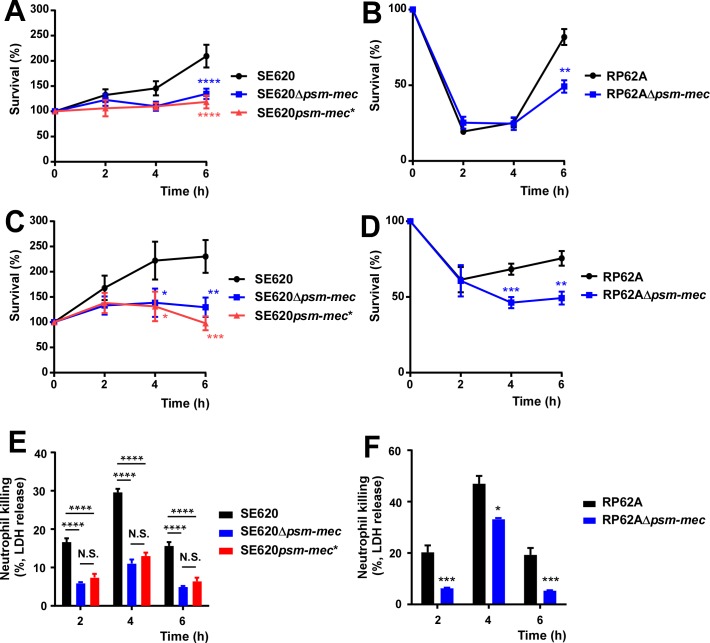
Bacterial survival during incubation with human neutrophils and in whole human blood. (**A,B**) Survival in whole, heparinized human blood. (**C,D**) Survival during incubation with human neutrophils (MOI 10:1). (**E,F**) Killing of human neutrophils (MOI 100:1). Error bars show ±SEM. *, *P* <0.05, **, *P* <0.01. ***, *P* <0.001. ****, *P* <0.0001 (unpaired t-tests for RP62A; 1-way ANOVA with Bonferroni post-tests for SE620). Δ*psm-mec*, isogenic *psm-mec* deletion mutant; psm-mec*, *psm-mec* gene start codon mutant. In (A) and (C), no comparisons between SE620Δ*psm-mec* and SE620psm-mec* were statistically significant.

In addition to being pro-inflammatory, the PSM-mec toxin has pronounced cytolytic capacity [12]. Cytolysis by PSMs is believed to be most important for infection when bacteria are engulfed in the phagosome of neutrophils and other phagocytes [16, 17]. Survival of bacteria when incubated with human neutrophils and survival in whole human blood was significantly higher with the *S*. *epidermidis* wild-type strain than with Δ*psm-mec* or psm-mec* start codon mutants, as was killing of neutrophils when incubated with whole bacteria (**Fig 4**), emphasizing the role of the PSM-mec toxin in evasion of neutrophil killing and resistance to the strong bactericidal capacities of immune defense mechanisms in human blood. Together, these results indicate that the both the pro-inflammatory and cytolytic capacities of the PSM-mec peptide contribute to the development of *S*. *epidermidis* sepsis.

**Fig 5:**
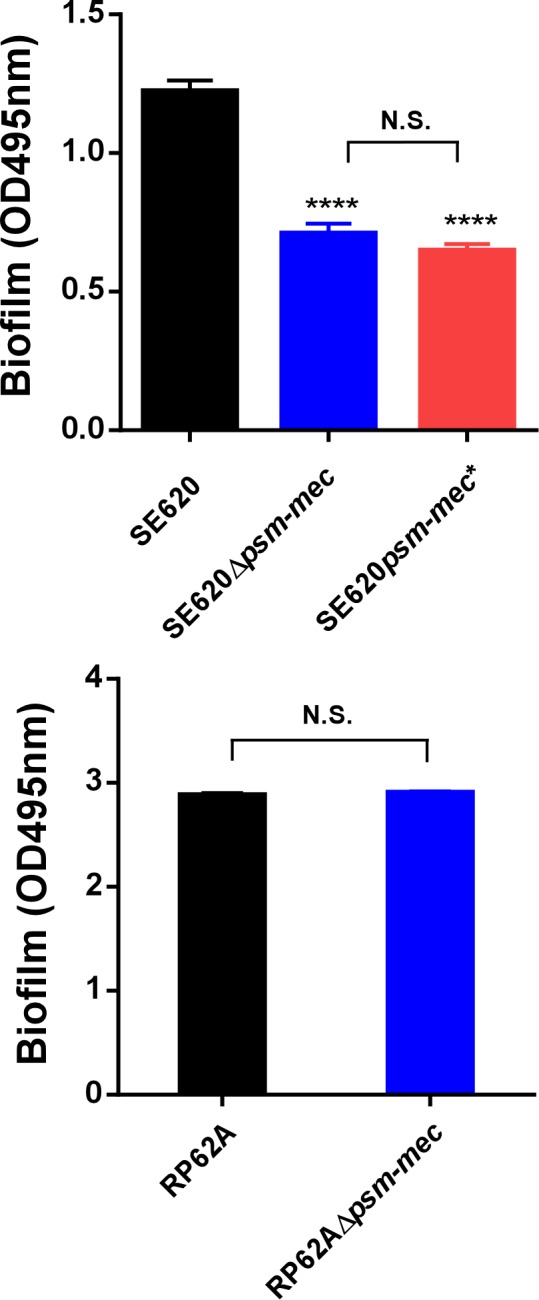
Impact of *psm-mec* on biofilm formation in *S. epidermidis*. Biofilm formation by *S*. *epidermidis* strains and isogenic *psm-mec* deletion and *psm-mec* start codon mutants. Biofilm formation was measured using a semi-quantitative microtiter plate assay. 24 wells per group were measured. ****, P<0.0001 (1-way ANOVA, Bonferroni post tests vs. wild-type strain); N.S., not significant. Error bars show ±SEM. Δ*psm-mec*, isogenic *psm-mec* deletion mutant; psm-mec*, *psm-mec* gene start codon mutant.

In *S*. *aureus*, the *psm-mec* locus has also been implicated in biofilm-forming capacity, although effects were generally minor and highly strain-dependent [12]. Similar to *S*. *aureus*, biofilm formation in *S*. *epidermidis* was affected only slightly by the *psm-mec* locus, and as this was seen only in one strain, similarly strain-dependent (**Fig 5**). In that strain, SE620, the effect was due to the PSM-mec peptide, not the *psm-mec* srRNA. These findings indicate that during indwelling medical device-associated blood stream infections by *S*. *epidermidis*, the impact of PSM-mec generally is by contributing to the development of sepsis, as we have shown here, rather than by promoting biofilm formation on the device itself.

**Fig 6:**
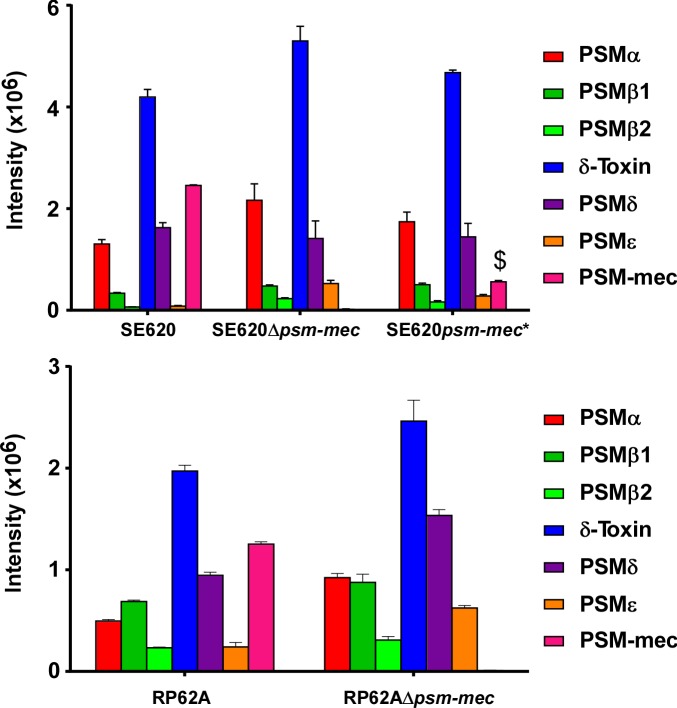
PSM production in *S*. *epidermidis* strains and isogenic *psm-mec* deletion and *psm-mec* start codon mutants. PSMs were measured in stationary phase (16 h) cultures using RP-HPLC/ESI-MS in triplicate. Error bars show ±SEM. $, Residual PSM-mec production in the SE620 *psm-mec* start codon mutant is likely due to strong gene expression and usage of a non-canonical start codon, as we previously found also in *S*. *aureus psm-mec* start codon mutants [14].

**Fig 7:**
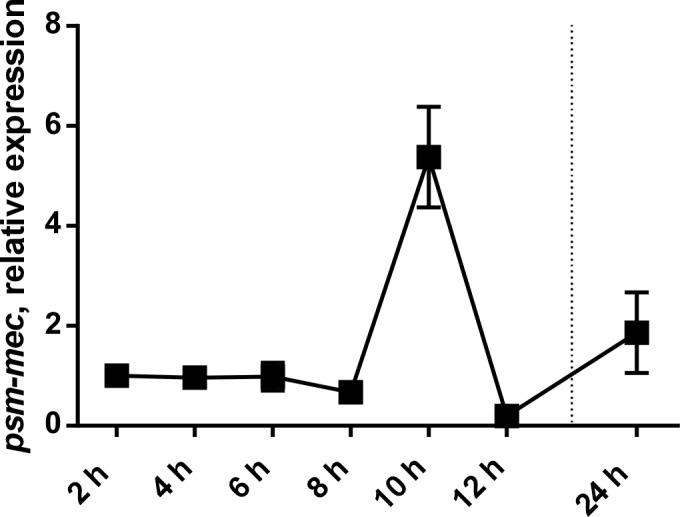
Time-dependent expression of *psm-mec*. Expression of the *psm-mec* RNA relative to that of 16S RNA in *S*. *epidermidis* RP62A during growth in TSB. The experiment was performed in triplicate. Error bars show ±SEM.

Our results showed that the *psm-mec* srRNA is not involved with sepsis or other relevant virulence phenotypes in *S*. *epidermidis*. As a previous study suggested that the *psm-mec* srRNA leads to gene regulatory changes in *S*. *epidermidis* [15], based on the introduction of a *psm-mec* expressing plasmid into *S*. *epidermidis*, we also directly investigated whether the *psm-mec* locus has a gene regulatory impact in *S*. *epidermidis*. The most important regulatory effect of the *psm-mec* locus in *S*. *aureus*, by which the sometimes negative impact of the *psm-mec* locus on virulence in *S*. *aureus* was explained, has been reported to consist in the alteration of the expression of other, core genome-encoded PSMs [18]. PSM expression was altered only to a very low extent in the *psm-mec*-negative as compared to the wild-type *S*. *epidermidis* strains, with changes only significant for some PSMs and never exceeding a factor of ~ 1.5 (**Fig 6**). This demonstrates that there is only a very minor effect of the *psm-mec* srRNA on PSM expression when analyzed directly in the *S*. *epidermidis* background. Furthermore, we analyzed genome-wide gene expression in the *psm-mec* mutants of both strains by microarray analysis (**Tables 1 and 2**). For microarray analysis, strains were grown to the maximum of PSM-mec expression as determined by qRT-PCR (10 h) (**Fig 7**). While we observed gene regulatory changes that were due to the *psm-mec* srRNA, they mostly comprised metabolic (e.g., riboflavin and purin/pyrimidine synthesis) rather than virulence genes, and were inconsistent between the two strains. Notably, the results of the previously claimed impact of the *psm-mec* locus on virulence would be negative [13, 15, 18], contrasting the positive effect we observed in the mouse sepsis model. Such a gene regulatory mechanism can thus be ruled out as underlying *psm-mec*-mediated development of *S*. *epidermidis* sepsis.

**Table 1:** Microarray results, RP62A^1^.

RP62A			
Gene	Number^2^	Function	RP62AΔ*psm-mec* vs. RP62A^3^
pyrB	SERP0766	aspartate carbamoyltransferase	18.14
ribE	SERP1327	riboflavin synthase subunit alpha	16.04
ribBA	SERP1326	3,4-dihydroxy-2-butanone-4-phosphate synthase	15.16
purM	SERP0656	phosphoribosylaminoimidazole synthetase	14.94
purF	SERP0655	amidophosphoribosyltransferase	14.77
purN	SERP0657	phosphoribosylglycinamide formyltransferase	14.72
serS	SERP2545	seryl-tRNA synthetase	13.55
purH	SERP0658	bifunctional phosphoribosylaminoimidazolecarboxamide formyltransferase/IMP cyclohydrolase	13.45
purD	SERP0659	phosphoribosylamine—glycine ligase	11.92
arcB-2	SERP2351	ornithine carbamoyltransferase	11.33
ribD	SERP1328	riboflavin biosynthesis protein RibD	10.99
-	SERP2546	hypothetical protein	9.88
ribH	SERP1325	6,7-dimethyl-8-ribityllumazine synthase	9.50
arcC	SERP2352	carbamate kinase	9.07
purL	SERP0654	phosphoribosylformylglycinamidine synthase II	7.33
-	SERP2381	NADH:flavin oxidoreductase/fumarate reductase, flavoprotein subunit	7.27
#N/A	SE2245		7.00
-	SERP2279	hypothetical protein	6.65
-	SERP2278	hypothetical protein	6.50
-	SERP2380	drug transporter	6.26
cysH	SERP2192	phosophoadenylyl-sulfate reductase	-95.16
sat	SERP2186	sulfate adenylyltransferase	-18.63
-	SERP0094	cysteine synthase/cystathionine beta-synthase family protein	-13.93
cysK	SERP0152	cysteine synthase	-9.84
-	SERP0095	trans-sulfuration enzyme family protein	-8.77
-	SERP2196	MarR family transcriptional regulator	-8.64
-	SERP1478	GntR family transcriptional regulator	-8.26
-	SERP2003	amino acid ABC transporter ATP-binding protein	-7.99
-	SERP2187	hypothetical protein	-7.65
-	SERP2195	alpha keto acid dehydrogenase complex, E3 component, lipoamide dehydrogenase	-7.46
cysC	SERP2185	adenylylsulfate kinase	-7.41
-	SERP2004	amino acid ABC transporter permease	-6.80
lacE	SERP1790	PTS system, lactose-specific IIBC components	-6.80
-	SERP1980	nitrite extrusion protein	-5.78
cysI	SERP2190	sulfite reductase subunit beta	-5.74
lacG	SERP1789	6-phospho-beta-galactosidase	-5.70
lacF	SERP1791	PTS system, lactose-specific IIA component	-5.07
-	SERP0056	hypothetical protein	-4.91
-	SERP2005	amino acid ABC transporter amino acid-binding protein	-4.86
lacA	SERP1795	galactose-6-phosphate isomerase subunit LacA	-4.81

^1^ The top 20 down- and up-regulated genes are shown.

^2^ Gene numbers are for strain RP62A, unless a specific gene is not annotated or exists in that strain, in which case the number for strain ATCC12228 is shown.

^3^Up-regulation is shown by positive numbers, down-regulation by negative numbers.

**Table 2:** Microarray results, SE620^1^.

SE620					
Gene	Number^2^	Function	SE620Δ*psm-mec* vs. SE620	SE620psm-mec* vs. SE620	SE620Δ*psm-mec* vs.SE620psm-mec*
-	SERP2245	tributyrin esterase EstA	4.25	1.24	3.42
ribE	SERP1327	riboflavin synthase subunit alpha	4.18	16.69	-3.99
ribBA	SERP1326	3,4-dihydroxy-2-butanone-4-phosphate synthase	3.62	12.67	-3.50
-	SERP2546	hypothetical protein	3.41	1.41	2.42
ribD	SERP1328	riboflavin biosynthesis protein RibD	3.06	10.33	-3.38
-	SERP0664	hypothetical protein	3.00	1.89	1.59
-	SERP2321	hypothetical protein	2.92	5.19	-1.77
rbsK	SERP2100	Ribokinase	2.82	2.29	1.23
ribH	SERP1325	6,7-dimethyl-8-ribityllumazine synthase	2.79	8.78	-3.15
purS	SERP0652	phosphoribosylformylglycinamidine synthase, PurS protein	2.76	2.95	-1.07
-	SERP1933	hypothetical protein	2.71	1.65	1.64
-	SERP2364	succinyl-diaminopimelate desuccinylase	2.66	3.38	-1.27
-	SERP2354	tributyrin esterase EstA	2.64	1.88	1.40
-	SERP1498	ammonium transporter	2.58	4.47	-1.74
-	SERP1933	hypothetical protein	2.53	1.63	1.55
mraY	SERP0747	phospho-N-acetylmuramoyl-pentapeptide-transferase	2.49	1.27	1.96
purQ	SERP0653	phosphoribosylformylglycinamidine synthase I	2.40	2.48	-1.03
-	SERP2357	amino acid ABC transporter permease	2.39	2.17	1.10
-	SERP1529	hypothetical protein	2.34	-1.90	4.46
purF	SERP0655	amidophosphoribosyltransferase	2.32	2.85	-1.23
-	SERP0473	hypothetical protein	-6.11	-1.43	-4.28
-	SERP0473	hypothetical protein	-5.46	-1.31	-4.17
-	SERP1474	hypothetical protein	-5.27	-11.93	2.26
-	SERP1478	GntR family transcriptional regulator	-4.32	-11.37	2.63
-	SERP2158	amino acid permease	-3.75	-3.27	-1.14
-	SERP0944	ThiJ/PfpI family protein	-3.71	-1.71	-2.16
trpG	SERP0938	anthranilate synthase component II	-3.65	-1.87	-1.96
-	SERP1475	ABC transporter ATP-binding protein	-3.46	-8.38	2.42
czrA	SERP1755	CzrA family transcriptional regulator	-3.30	-2.32	-1.42
-	SERP0273	alpha/beta hydrolase	-3.08	-1.73	-1.78
-	SERP0507	CBS domain-containing protein	-3.06	-1.48	-2.07
-	SERP2091	hypothetical protein	-2.94	-1.32	-2.22
-	SE0735		-2.91	-1.12	-2.60
-	SERP1476	hypothetical protein	-2.91	-9.68	3.33
-	SERP1477	ABC transporter ATP-binding protein	-2.89	-9.52	3.30
-	SERP0620	hypothetical protein	-2.80	-1.04	-2.69
-	SERP2129	short chain dehydrogenase/reductase family oxidoreductase	-2.78	-1.28	-2.17
-	SE0082		-2.71	-1.12	-2.42
-	SERP0385	ABC transporter ATP-binding protein	-2.69	-1.56	-1.73
-	SERP1479	hypothetical protein	-2.66	-2.94	1.11

^1^ The top 20 down- and up-regulated genes are shown. Sorting was by the SE620Δ*psm-mec* vs. SE620 comparison and the gene expression changes for the same genes for the other comparisons are shown.

^2^ Gene numbers are for strain RP62A, unless a specific gene is not annotated or exists in that strain, in which case the number for strain ATCC12228 is shown.

^3^Up-regulation is shown by positive numbers, down-regulation by negative numbers.

Our results may explain the highly inconsistent phenotypes that have been attributed to *psm-mec* in *S*. *aureus* [11–13], inasmuch as the *psm-mec* locus may exert effects in the background of *S*. *aureus* strains that differ from its original role in the CNS environment. One such possibility that remains to be investigated is that the highly expressed *psm-mec* mRNA interferes with other DNA or RNA sequences in *S*. *aureus*. Furthermore, the *psm-mec* srRNA barely exceeds the limits of the *psm-mec* gene [14], which contrasts the only other case of an srRNA with an embedded peptide toxin in staphylococci, namely the well-described regulatory RNAIII of the staphylococcal accessory gene regulator (Agr) system. RNAIII significantly exceeds the boundaries of the embedded PSM peptide gene, *hld* [19]. Together, these observations suggest that the *psm-mec* srRNA does not serve a well-defined general purpose in virulence gene regulation.

In conclusion, our study reveals that sepsis due to MRSE is mediated to a large extent by the PSM-mec peptide toxin, representing the first example of a toxin being made responsible for the development of CNS sepsis. Our study was largely based on the investigation of isogenic *psm-mec* mutants in clinical strains of *S*. *epidermidis*, using tissue culture and animal infection models. Future clinical work is needed to assess whether PSM-mec and/or other toxins contribute to sepsis in humans. Importantly, our results suggest that CNS sepsis may be amenable to virulence-targeted therapeutic approaches, such as those targeting the quorum-sensing system Agr [20], which strictly regulates PSM expression [21], or monoclonal antibody-based therapy directed against the toxin.

## Methods

### Bacterial strains and growth conditions

Strain RP62A is a genome-sequenced clinical MRSE isolate [22]. Strain SE620 is an MRSE clinical isolate from Norway [23]. Isogenic Δ*psm-mec* deletion mutants and the psm-mec* start codon mutant were produced with the constructs previously used for *S*. *aureus* [12, 14], using a strategy with the allelic exchange vector pKOR1 [24]. The *psm-mec* locus and adjacent DNA do not differ between *S*. *aureus* and *S*. *epidermidis* [25]. For construction of the psm-mec* mutant, the start codon mutation was created by introducing a ClaI restriction site (introducing ATC instead of the ATG start codon) using primer PSMEClarev GAGGGTATGCATATCGATTTCACTGGTGTTATTACAAGC and primer PSMECladir (reverse complement of PSMEClarev). Two PCR fragments were amplified using those primers and primers psmEatt1 and psmEatt2, respectively [12], cut with ClaI, ligated, and cloned into pKOR1. The resulting plasmid was used for allelic replacement as described [24]. Growth patterns of the mutants were indistinguishable from those of the wild-type (**S3 Fig**). Strains were grown in tryptic soy broth (TSB), unless otherwise noted.

### Mouse sepsis model

Female, 6–10 weeks old, C57BL/6NCRl (Charles River) mice were used. The mice were injected via the tail vein with 5 x 10^8^ CFU in 100 μl phosphate-buffered saline (PBS) of the indicated bacterial strains grown to mid-exponential growth phase and monitored for disease development every 8 h for up to 120 h. This dosis was determined to be minimally necessary to achieve mortality and production of inflammatory cytokines (**S4 Fig**). Animals were euthanized immediately if showing signs of respiratory distress, mobility loss, or inability to eat and drink. Cytokine concentrations were measured at 2 and/or 12 h, as indicated, using commercially available ELISA kits (IL-1β, TNF-α, BD BioSciences; CXCL1, R&D Systems).

### Interaction of bacteria with human neutrophils and bacterial survival in human blood

For survival in whole blood experiments, about 10^8^ bacteria in 100 μl Dulbecco’s PBS from mid-exponential growth phase were added to 500 μl heparinized human blood and mixtures were incubated for 6 h. Aliquots were taken at 2-h intervals, and CFU were determined by plating and incubating plates overnight at 37**°**C.

For neutrophil interaction experiments, neutrophils were isolated from the venous blood of human volunteers as described [26]. Bacteria from mid-exponential growth phase were mixed with neutrophils at an MOI (bacteria/neutrophils) of 10:1. Bacteria/neutrophil mixtures were incubated at 37**°**C, 5% CO_2_, 90% humidity for 6 h. At 2-h intervals, 50 μl of Triton X-100 was added to the 200-μl bacteria/neutrophil suspensions, aliquots were plated, and plates incubated at 37**°**C overnight for CFU counting. Alternatively, the rate of neutrophil lysis promoted by the bacteria was determined after 4–h incubation using a lactate dehydrogenase (LDH) assay at an MOI of 100:1.

### Biofilm formation

Biofilm formation was assessed in a semi-quantitative 96-well microtiter plate assay as previously described [27], using TSB + 0.5% glucose.

### PSM measurement

Relative PSM concentrations in culture filtrates were determined as described using reversed-phase high-pressure liquid chromatography/electrospray mass spectrometry (RP-HPLC/ESI-MS) [28].

### Quantitative real-time (RT)-PCR and microarray analysis

Quantitative RT-PCR was performed as previously described [29] with the following oligonucleotides: psm-mecF, TGCATATGGATTTCACTGGTGTTA, psm-mecR, CGTTGAATATTTCCTCTGTTTTTTAGTTG, psm-mec probe, ATTTAATCAAGACTTGCATTCAG. Expression was measured relative to that of 16S RNA. Cultures were grown to the maximum of *psm-mec* expression as determined by qRT-PCR (10 h). Total RNA and cDNA were prepared as described [30]. Biotinylated *S*. *aureus* cDNA was hybridized to custom Affymetrix GeneChips (RMLChip 3) with 100% coverage of chromosomal genes from strains *S*. *epidermidis* RP62A and scanned according to standard GeneChip protocols (Affymetrix). Each experiment was replicated 3 times. Affymetrix GeneChip Operating Software was used to perform the preliminary analysis of the custom GeneChips at the probe-set level. Subsequent data analysis was performed as described [30]. The complete set of microarray data was deposited in NCBIs Gene Expression Omnibus (GEO, http://www.ncbi.nlm.nih.gov/geo/) and is accessible through GEO Series accession number GSE85265.

### Analysis of mRNA stability

To determine *psm-mec* mRNA stability in strains *S*. *epidermidis* SE620 and the psm-mec* start codon mutant, bacteria were cultured at 37**°**C for 10 hours. At time t = 0 min, rifampicin (50 mg/ml stock in DMSO) was added to the cultures to a final concentration of 100 μg/ml. One-ml aliquots were taken and immediately centrifuged at 4**°**C to pellet cells, which were then frozen at -70**°**C. Remaining cultures were further incubated at 37**°**C with shaking; one-ml aliquots were taken at the indicated times and RNA was subsequently isolated from all cell pellets as described [29]. Samples were analyzed by qRT-PCR using primers psm-mecR and psm-mecF with a SuperScript III Platinum SYBR Green One-Step qRT-PCR kit (Invitrogen) according to the manufacturer’s instructions. Expression was measured relative to that of 16S RNA.

### Statistics

Statistical analysis was performed using GraphPad Prism Version 6.0. Comparisons were by 1-way or 2-way ANOVA for comparisons of three and more, and by unpaired t-tests for comparisons of 2 groups. Error bars show ±SEM.

### Ethics statement

The animal protocol (LB1E) was reviewed and approved by the Animal Care and Use Committee at the NIAID, NIH, according to the animal welfare act of the United States (7 U.S.C. 2131 et. seq.). All mouse experiments were performed at the animal care facility of the NIAID, Building 50, in accordance with approved guidelines. All animals were euthanized by CO_2_ at the end of the studies. Human neutrophils were isolated from blood obtained under approved protocols at the NIH Blood Bank or with a protocol (633/2012BO2) approved by the Institutional Review Board for Human Subjects, NIAID, NIH. All subjects were adult and gave informed written consent.

## Supporting Information

S1 Fig PSM-mec production in clinical MRSE strains. A clinical strain collection was analyzed for PSM-mec production by RP-HPLC/MS of stationary-phase culture filtrates. The horizontal line shows the mean. Colored dots show the production in the strains used in this study.
(TIF)

S2 Fig Stability of *psm-mec* and psm-mec* RNA. Data are not significantly different between the two groups at any time point.
(TIF)

S3 Fig Growth curves in TSB of *S*. *epidermidis* strains and isogenic *psm-mec* deletion and *psm-mec* start codon mutants. (TIF)

S4 Fig Determination of the minimal dose in the mouse infection model. (**A,B**) Mortality in the mouse bacteremia model at different doses of strains SE620 and RP62A, respectively. (**C**) Concentration of the inflammatory cytokine CXCL-1 (TNF-α) in mouse blood at 12 h. Note only one mouse could be used for the group infected with strain SE620, as the others died very early. (**A-C**) n = 5 in every group.
(TIF)
