# Supplementary figures and images for: Toxin Mediates Sepsis Caused by Methicillin-Resistant Staphylococcus epidermidis
Source: PLoS Pathog. 2017 Feb 2;13(2):e1006153. doi: 10.1371/journal.ppat.1006153 (PMC5289634; doi:10.1371/journal.ppat.1006153)

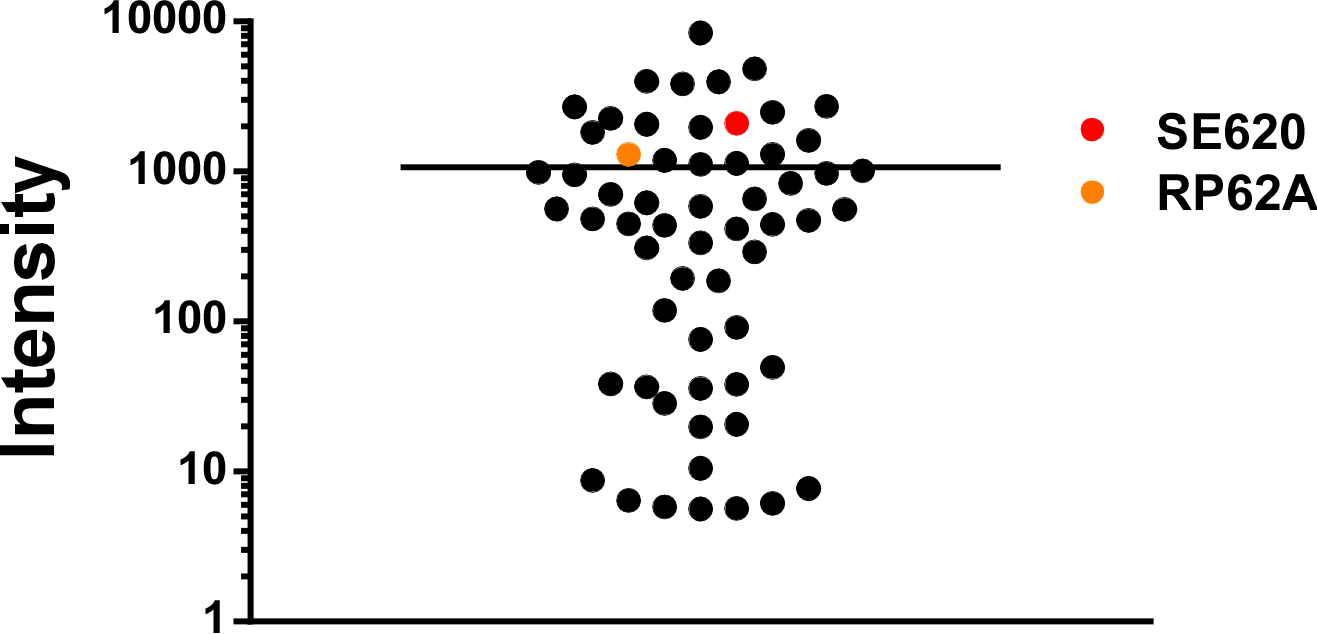

Supplement: S1 Fig — A clinical strain collection was analyzed for PSM-mec production by RP-HPLC/MS of stationary-phase culture filtrates. The horizontal line shows the mean. Colored dots show the production in the strains used in this study. (TIF) [file ppat.1006153.s001.tif]

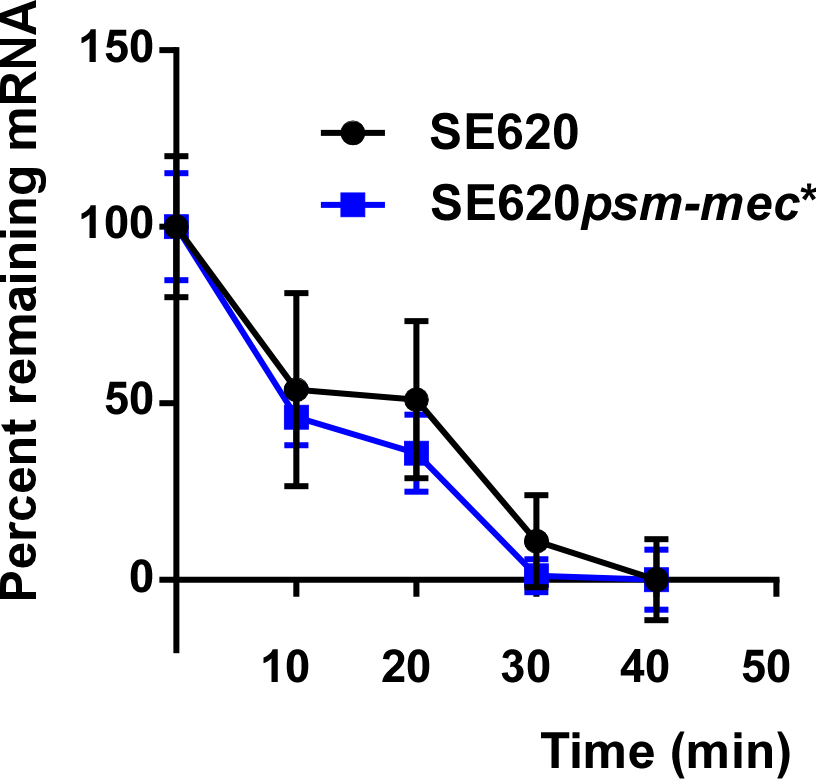

Supplement: S2 Fig — Data are not significantly different between the two groups at any time point. (TIF) [file ppat.1006153.s002.tif]

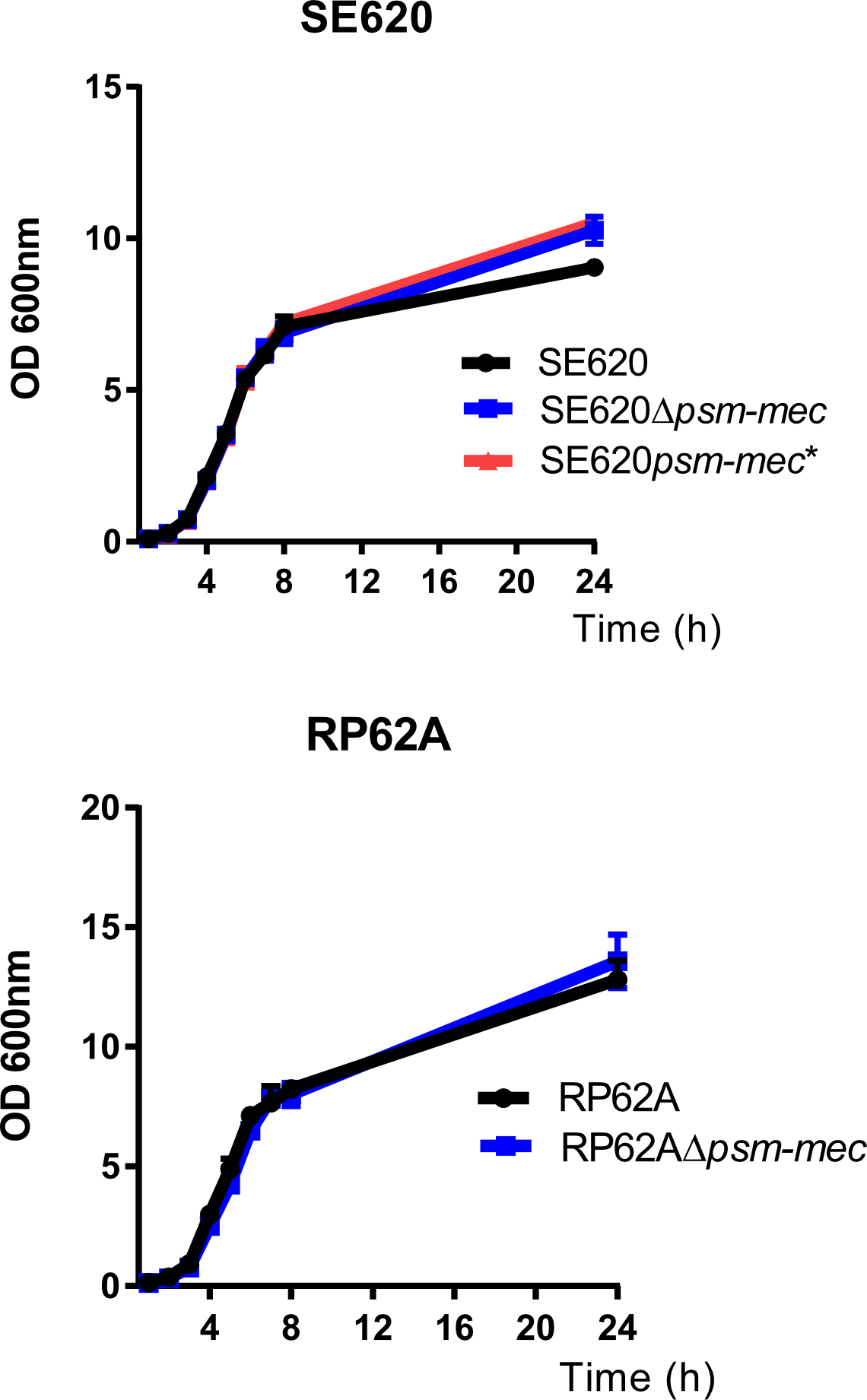

Supplement: S3 Fig — (TIF) [file ppat.1006153.s003.tif]

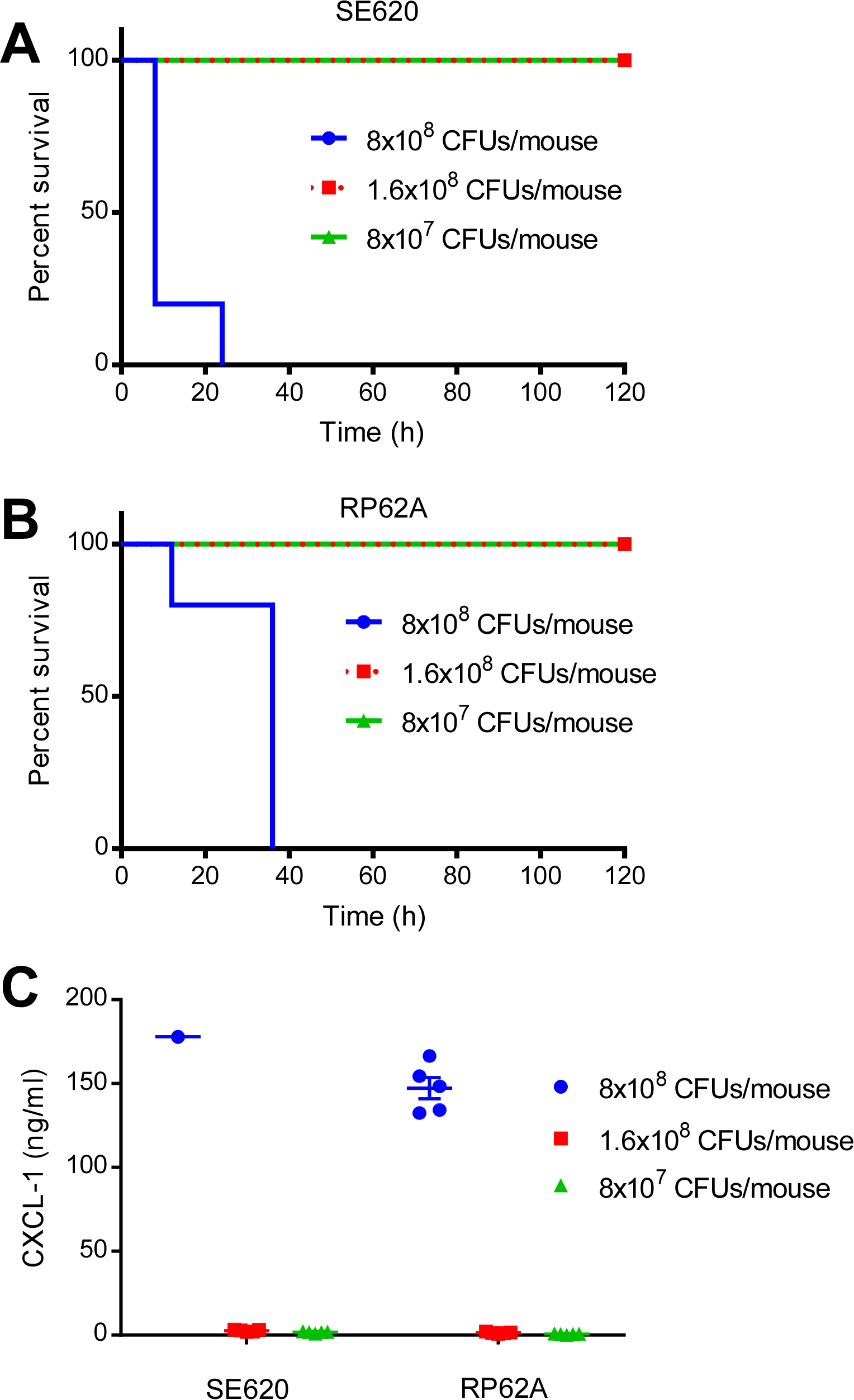

Supplement: S4 Fig — (A,B) Mortality in the mouse bacteremia model at different doses of strains SE620 and RP62A, respectively. (C) Concentration of the inflammatory cytokine CXCL-1 (TNF-α) in mouse blood at 12 h. Note only one mouse could be used for the group infected with strain SE620, as the others died very early. (A-C) n = 5 in every group. (TIF) [file ppat.1006153.s004.tif]
